# 2D and 3D Erosion Landscape Analysis of Endodontic-Treated Teeth Using EDTA and HEDP as Chelating Agents: A High-Resolution Micro-Computed Tomographic Study

**DOI:** 10.3390/dj11120286

**Published:** 2023-12-12

**Authors:** Parichehr Zarean, Michael Göllner, Paridokht Zarean, Klaus W. Neuhaus

**Affiliations:** 1 Department of General Pediatric and Adolescent Dentistry, University Center for Dental Medicine Basel UZB, University of Basel, Mattenstrasse 40, 4058 Basel, Switzerland; parichehr.zarean@unibas.ch (P.Z.); paridokht.zarean@unibas.ch (P.Z.); 2 F. Hoffmann-La Roche Ltd., Grenzacherstrasse 124, 4070 Basel, Switzerland; michael.goellner@roche.com; 3 Department of Restorative, Preventive and Pediatric Dentistry, School of Dental Medicine, University of Bern, Freiburgstrasse 7, 3010 Bern, Switzerland

**Keywords:** ethylenediaminetetraacetic acid, HEDP, micro-CT, smear layer, root canal preparation, therapeutic irrigation

## Abstract

This study aimed to assess the amount of erosion during activated endodontic irrigation with either HEDP or EDTA via high-resolution micro-computed tomography. Two root canals of twenty premolars were prepared with ProTaper Next and irrigated with sodium hypochlorite. Palatal canals, which served as control groups, were sealed, while buccal canals were further irrigated with either EDTA (n = 10) or HEDP (n = 10), which served as test groups. Micro-CT was performed to measure erosion depth. For 2D and 3D measurements, non-parametric repeated ANOVA measurements and post hoc tests were performed. 2D analysis showed highly significant differences between the case groups at each position of the root (*p* ≤ 0.01). The cervical and apical positions showed significant differences in the EDTA group (*p* = 0.03). The 3D analysis also showed significant differences between both chelating agents (*p* < 0.01) and the case and control groups (*p* = 0.01). The mean erosion depths in the cervical, middle, and apical thirds of the EDTA group were 45.75, 41.79, and 32.25 µm, and for the HEDP group were 20.25, 16.40, and 15.96 µm, respectively. HEDP seems to have a significantly less erosive effect. Different irrigation protocols with harsher conditions, as might be the case during endodontic retreatment, could be assessed with micro-CT.

## 1. Introduction

Chemomechanical root canal treatment involves both mechanical instrumentation and the enlargement of root canals, coupled with endodontic irrigation [1,2,3]. The primary objectives of endodontic irrigation are to dissolve organic tissue, disinfect the root canals, prevent or address periapical infections, and clean regions that may be inaccessible to instruments [4,5].

Due to its ability to dissolve vital or necrotic soft tissues and its strong antimicrobial capacities, sodium hypochlorite (NaOCl) is regarded as the first-choice endodontic irrigation solution [6,7]. However, it does not show a chelating or demineralizing effect [5,8].

Following the instrumentation of root canal walls, the process generates debris comprising mineralized and organic tissue. The accumulation of this debris in recesses, accessory canals, or as a thin layer on root canal dentin, known as the smear layer, can impede effective cleaning of the endodontic system [3]. The smear layer comprises both inorganic and organic components [9,10,11]. Because NaOCl is ineffective in removing the inorganic component of the smear layer, the use of a chelating agent or acid has been recommended as an additional irrigation solution [9,10]. In recent years, various materials, including ethylenediaminetetraacetic acid (EDTA) [4,12,13,14], etidronic acid or 1-hydroxyethylidene 1,1-diphosphonic acid (HEDP) [8,15], and organic acids such as citric acid [16,17], polyacrylic acid [18,19], tannic acid [20,21], and lactic acid [22], have been explored for their efficacy in removing the smear layer to enhance the cleaning of the endodontic system. The use of these acids during endodontic irrigation has the potential to induce erosion in root dentine [3].

It has been proposed that rinsing with 17% EDTA as a chelating agent during root canal instrumentation can potentially eliminate and/or prevent the formation of the smear layer [3,9]. Nonetheless, the use of EDTA comes with certain drawbacks. In the combination of EDTA and NaOCl, the free available chlorine is reduced, leading to a potential decrease in the tissue dissolution and antimicrobial efficacy of NaOCl. Consequently, it is recommended to follow two separate irrigation procedures [23,24,25,26]. EDTA exhibits restricted antibacterial activity and a limited ability to remove the smear layer in the apical thirds of the root canal. Furthermore, certain studies have reported dental erosion associated with the use of EDTA [8,27,28]. Consequently, alternative chelating agents have been explored as potential substitutes for EDTA in recent research.

Recently, 1-hydroxyethylidene 1,1-diphosphonic acid (HEDP) has been introduced as a potent chelating agent for endodontic irrigation, demonstrating effective demineralization of the inner surface of root canals. HEDP holds promise as a potential alternative to the presently employed irrigations, such as sodium hypochlorite and a final rinse with 17% EDTA. Notably, HEDP exhibits stability even when dissolved in sodium hypochlorite, remaining active for up to 1 h [15,29]. One notable advantage of this material is its dual functionality in tissue dissolution and smear layer removal throughout the entire process of cleaning and shaping root canals in root canal treatments. This dual action has the potential to augment the antimicrobial efficacy of NaOCl by preventing the formation of the smear layer during the treatment procedure [3,24,30,31,32]. This observation aligns with studies by Neelakantan et al. and Morago et al., suggesting that continuous chelation by HEDP can enhance the disinfecting efficacy of NaOCl in infected root canals [32,33]. It is important to note, however, that the addition of NaOCl increases the erosive effect of HEDP on dental root walls [4].

In a comparative assessment of these chelating agents, Kfir et al. [3] examined the impact of HEDP and EDTA in combination with NaOCl on the cleanliness and erosion of root canal walls using syringe and needle irrigation. Their findings revealed no significant differences. However, in a study conducted by Tartari et al., a milder effect of HEDP was observed [34].

Several investigations have aimed to quantify the extent of erosion within root canals. Methodologically, the majority of these studies have employed SEM and light microscopy to examine the eroded surface of root canal dentine walls [3,35,36,37]. Additionally, some studies have used fractured specimens to measure the actual depth of erosion [38,39]. However, these methods have their limitations. SEM may introduce a degree of subjectivity, and fractured specimens only permit the evaluation of a single region within an entire root [11].

Micro-computed tomography (micro-CT) stands out as a non-invasive technique for the in vitro three-dimensional evaluation of erosion within root canals. This method facilitates precise assessment by generating high-accuracy and high-resolution images both before and after interventions, all without the need for sample destruction [40,41].

To the authors’ knowledge, there is a lack of information addressing the severity and depth of erosion inside the root canal walls upon root canal treatments with the activation of irrigation [42], and this is the first study that compared the severity and depth of erosion with a non-destructive high-resolution micro-CT method. Therefore, this study aimed to assess the amount of root dentine erosion during ultrasonically activated endodontic irrigation with either HEDP or EDTA by using a non-destructive high-resolution micro-CT, which enabled us to assess the whole root canal and not only the single region of the root upon fracture. The null hypothesis posited that there exists no disparity in the severity of dentine erosion between these two chelating agents.

## 2. Materials and Methods

### 2.1. Tooth Selection

In this in vitro study, twenty upper-first human premolars were selected, which were extracted for different reasons. The teeth were irreversibly anonymized and stored in a 1% chloramine solution container. The inclusion criteria were complete root formation with two separate roots, no fillings, cavitated caries lesion, no root caries or signs of enamel or dentine hypoplasia, and no visible fractures. According to the following formula, the sample size was calculated, considering α = 0.05 and β = 0.2 with a study power of 80%.

$$n=\frac{2\sigma^{2}{\left(z_{1-\alpha /2}+z_{1-\beta }\right)}^{2}}{\delta^{2}}$$

### 2.2. Sample Preparation

After preparing an access cavity, straight-line access to the two orifices was established. The working length was established using an ISO 10 hand instrument (DentsplyMaillefer, Ballaigues, Switzerland) as 0.5 mm short of the length that was reached when its tip was visible at the apical foramen.

The exposure of the root canals was conducted with the ProTaper Next system using sequences X1 to X4 (Dentsply Sirona, Tulsa, OK, USA) in an Xsmart motor (Maillefer, Ballaigues, Switzerland) with the respective torque settings. The irrigation solution was 3% *w*/*v* sodium hypochlorite. Between changing the instruments, the canals were manually irrigated with 2 milliliters (mL) of sodium hypochlorite using a syringe with an endodontic irrigation probe (Max-i-Probe, 30G, Dentsply, Tulsa, OK, USA). The final irrigation was 5 mL of sodium hypochlorite [11].

The roots were then divided into the following groups: the palatal roots served as the control groups and the buccal roots were divided into two test groups including HEDP (n = 10) and EDTA (n = 10). During further irrigation of the buccal canals, the orifice of the palatal canal was tightly sealed with at least 5 mm Coltosol F (Coltène/Whaledent AG, Altstätten, Switzerland) to prevent the entry of irrigation solution. The irrigation protocol for the EDTA group was manual irrigation of 4 × 2 mL 17% *w*/*v* EDTA (Pulpdent, Waterwton, MA, USA) with a 4 × 30 s activation in between (IrriS, VDW, Munich, Germany with the VDW Ultra motor at 20%). The IrriS tip was introduced 1 mm short of the working length. Other test roots were irrigated with HEDP (Dual Rinse^®^ HEDP, Medcem, Weinfelden, Switzerland). The roots were then dried with paper tips and stored in tightly locked containers with 100% humidity.

### 2.3. Micro-CT

The teeth were analyzed using a SkyScan 1272 high-resolution desktop micro-CT (Bruker, Antwerp, Belgium) at a voltage of 80 kV, a current of 125 μA, and an isotropic voxel size of 2.25 μm. Samples were rotated through 180° and X-ray images were acquired at rotation steps of 0.05° using a 1200 ms exposure time through a 1 mm aluminum filter with a frame averaging of 6. Cross-section images were then reconstructed from the tomography projection images using the SkyScan NRecon V.2.0 (Bruker, Antwerp, Belgium) software program. 

### 2.4. 2D Measurements

**Figure 1:**
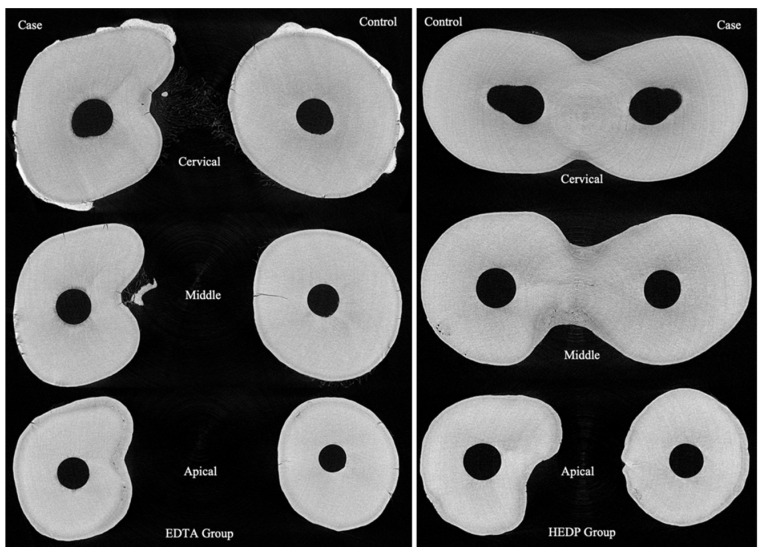
The middle layer of the cervical, middle, and apical thirds from two teeth in the EDTA and HEDP groups.

Roots were divided into three sections: cervical, middle, and apical (Figure 1). A middle layer within each section was randomly selected and spatially calibrated. Erosion depth (μm) was measured at twelve points per layer using ImageJ software Version 1.46r (U.S. National Institutes of Health, Bethesda, MD, USA). These points were evenly distributed in a circular pattern around the root canal, roughly 30 degrees apart.

### 2.5. 3D Measurements

**Figure 2:**
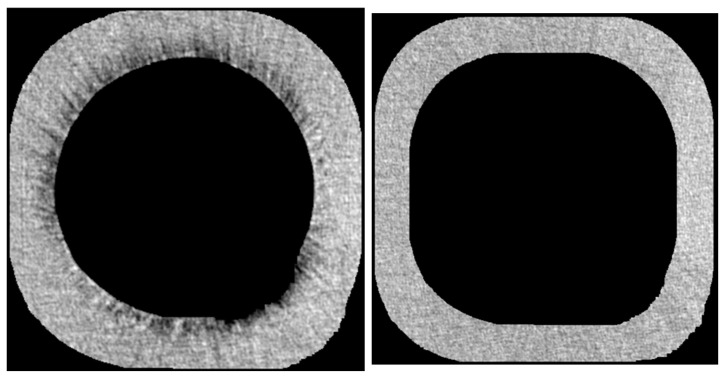
One layer of the selected ROI (**left**) and the reference region (**right**) in the EDTA case root.

VGStudio MAX 2.1 (Volume Graphics GmbH, Heidelberg, Germany) was used to select the region of interest (ROI) and reference region in all data generated from SkyScan NRecon. It was used in all roots, including EDTA and HEDP groups as well as control groups. The ROI, a sleeve with a depth of 50 voxels around the root canal, was selected to calculate erosion. The reference region consisted of a sleeve with a 50-voxel depth above the inner sleeve. (Figure 2) For image processing and to obtain density curves for each tooth, Python (Version 3.11.2, Python Software Foundation) was applied.

### 2.6. Statistics

All analyses were conducted with the statistical software R, version 4.0.2 [43]. For 3D analysis, the area under the obtained density curve as from the µCT was calculated. A characteristic density over the whole area under the curve (AUC)—named AUC-density—was defined as the ratio of the calculated AUC divided by the area under the curve if the density was constantly 1. This is not equivalent to calculating the mean density out of all calculated density points as it is a smoother and more robust approach.

A non-parametric repeated-measures ANOVA [44] was then used to assess the impact of repeated measurement factor positions (cervical, middle, apical) and groups (case, control) together with the independent factor, chelating agents (HEDP, EDTA), on both 2D and 3D outcomes. If the ANOVA found significant associations, post hoc tests in the form of Mann–Whitney tests or another repeated-measures ANOVA were performed to further investigate which sub-groups significantly differed. Throughout, *p*-values less than 0.05 were considered statistically significant. 

Post hoc tests were performed to check for group-wise differences. Post hoc tests were only performed for significant factors and *p*-values were thereby factor-wisely corrected via the method of “Holm”. Note that within control groups, only the effect of the chelating agent was tested to reduce the number of post hoc tests.

## 3. Results

### 3.1. 2D Measurements

#### 3.1.1. Descriptive

The mean erosion depths in μm per position and group are summarized in Table 1.

The results showed that the EDTA group had the highest mean of erosion depth. It was also obvious that values were highest for the cervical position, followed by the middle and then the apical position. Furthermore, the mean erosion of control groups showed no statistically significant differences.

#### 3.1.2. Statistical Analysis

**Figure 3:**
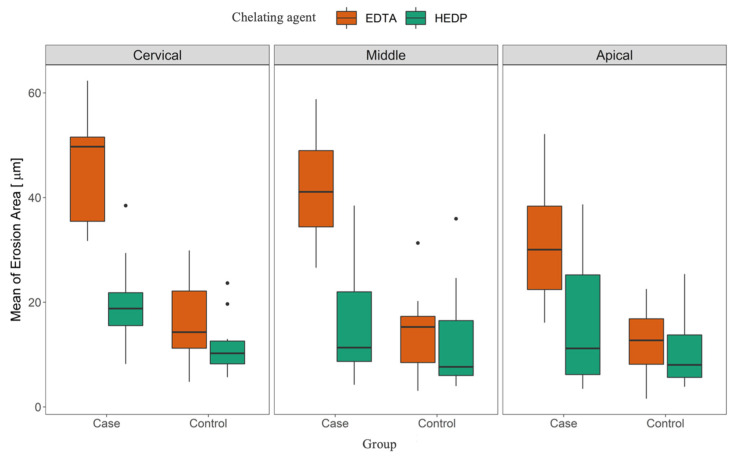
Mean erosion area grouped by position (cervical, middle, apical), group (case, control), and chelating agent (EDTA, HEDP).

The repeated-measures (RM) ANOVA showed that there was a clear difference between EDTA and HEDP groups overall (*p* = 0.01) and that there was evidence that the case groups significantly differed from the control groups (*p* < 0.01). It also showed that position affected erosion, as already mentioned in the descriptive section (*p* = 0.01) (Figure 3).

Post hoc pairwise comparisons with *p*-value correction via the “Holm” method showed that all comparisons of case and control were highly significant for both the EDTA and HEDP groups and at each position (all *p* ≤ 0.01 after correction). Both EDTA and HEDP significantly differed at each position in their case groups (all *p* ≤ 0.046 after correction). When comparing positions, the cervical and apical positions showed significant differences for the EDTA group (*p* = 0.03 after correction) (Table 2). Conversely, there was no significance found when comparing the position in the control group (*p* ≥ 0.84 after correction). This caused a significant interaction, as discussed above.

### 3.2. 3D Measurements

#### 3.2.1. Descriptive

**Figure 4:**
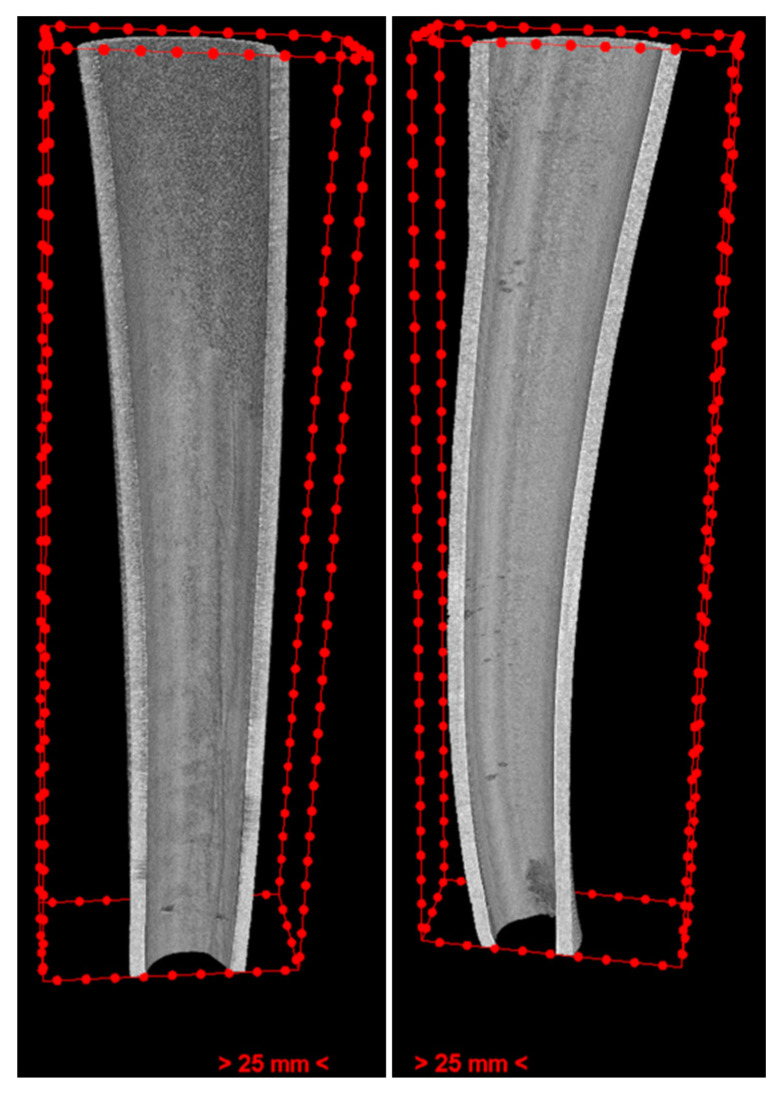
The inner cut of the case (**left**) and control (**right**) roots in the EDTA group, showing reduced erosion from the cervical to the apical in the case root. Furthermore, erosion depth is depicted on the cutting side.

The results showed clear differences between EDTA and HEDP in both the case and control groups. The median values were quite comparable in terms of overall positions for the HEDP group, whereas we see that the density became higher from the cervical to apical position for the EDTA group (Figure 4).

#### 3.2.2. Statistical Analysis

The repeated-measures (RM) ANOVA showed that there was a clear difference between EDTA and HEDP overall (*p* < 0.01). The same held for the significant effect of the groups (Case/Control) (*p* = 0.01). On the other hand, position failed to have a significant impact, but showed a very small, yet non-significant *p*-value (*p* = 0.055) when interacting with the chelating agents (Table 3). As stated in the descriptive section, densities tended to increase in the EDTA group while they remained stable in the HEDP group.

Post hoc pairwise comparisons with *p*-value correction via the “Holm” method showed that all comparisons regarding chelating agents were significant (all *p* ≤ 0.02 after correction). Without the visible EDTA outlier in the control group, the *p*-values would be even smaller. On the other hand, there were significant differences between the case and control roots for the EDTA group in the cervical (*p* = 0.04) and middle positions (*p* = 0.048), but not on the apical side (*p* = 1.00, all after correction). Considering the HEDP acid, we surprisingly detected a significant difference at the apical position (*p* = 0.02 after correction). Here, the differences were obviously on a very tiny scale and their practical relevance is very questionable.

## 4. Discussion

This study utilized non-destructive high-resolution micro-CT to assess the severity of root dentine erosion during activated endodontic irrigation with HEDP or EDTA.

The null hypothesis, which stated no difference in erosion severity between EDTA and HEDP, was rejected. Significant associations were observed between chelating agents, case and control groups, and positions within each third of the root canals with the 2D outcome mean erosion depth. Additionally, an interaction chelating agent group was found, as the case–control effect was much stronger for the EDTA group compared with the HEDP group. Furthermore, significant differences between the case and control roots were observed. Erosion was significantly stronger in the EDTA group for the case roots, but there was no significant difference in the control roots.

Regarding the 3D outcome AUC density, significant interactions were found for chelating agents and groups. Position showed no significant effect, though its interaction with chelating agents was only nearly insignificant. Additionally, significant differences between chelating agents were observed in both the case and control groups across all positions. The presence of an outlier in the control root of the EDTA group could be attributed to the restricted penetration of EDTA into the control roots during ultrasonic activation [45]. 

The higher level of collagen in the predentin layer may be another reason for enhanced erosion in younger teeth. We tried to take this into account using the same tooth as a test/control model. However, differences in root canal preparation, e.g., different amounts of brushing strokes during circumferential filing, could introduce bias, especially in younger teeth [45].

One of the strengths of our study is that it considered two roots of one tooth as control and case groups, because the hardness and biomechanical behavior of the roots may vary depending on the thickness of the dentinal wall, density of dentinal tubules, and the amount of intertubular dentine [46,47]. Another strength of this study is the approach to 3D measurements. Various initial attempts were made to automate these depth measurements, and the method utilizing AUC density emerged as the most suitable for semi-automated examination of the entire root.

Past research has not extensively addressed the erosion parameter, leading to a scarcity of available studies on this aspect [15,29,32]. Consequently, there is a paucity of literature available that aims to investigate erosion in 2D and 3D while focusing on the severity and position of erosion through a non-invasive method without destroying samples, which is an important difference between this study and the previous ones.

Our results agree with the results of Kfir et al. and Tartari et al., who found milder demineralized dentin using HEDP compared with EDTA [3,34].

Yadav et al. [8] also reported the lower efficacy of etidronic acid in removing the smear layer compared with SmearClear and MTAD, which could be associated with its weaker chelating effect [48], thereby resulting in less erosion.

Ulusoy et al. [4] showed peritubular and intertubular erosion upon the use of HEDP (9% etidronic acid) with or without 2.5% NaOCl, which agrees with the results of our study by having a level of erosion potentiated by NaOCl.

In our investigation, the dentinal wall density exhibited an ascending trend from the cervical to the apical part of the root canal within the EDTA group. This observation aligns with findings in existing studies and may be related to the larger diameter of the coronal part of the canal. This larger diameter facilitates the removal of the smear layer, potentially enhancing the effectiveness of the activation system [49,50]. The lower effect of EDTA as the chelating agent in the apical third is consistent with the findings reported by Fraser [51].

Elbahary et al. [11] utilized quantitative 3D surface texture analysis to investigate the erosive impact of NaOCl and EDTA administered in various sequences. The study employed a high-resolution confocal-disc-scanning measuring system for this purpose. They showed that NaOCl and EDTA increased the roughness of the inner surface of root canals when used as irrigating solutions, but the exact sequence of irrigation had no significant effect when applying NaOCl after EDTA revealed a higher roughness [11]. This aligns with the findings of Wang et al. [52], where erosion was quantified by measuring the atomic percentage via energy-dispersive X-ray spectroscopy. Their study demonstrated a high erosive effect of NaOCl when employed as the final irrigation solution [52]. In our investigation, we deliberately opted for a specific protocol involving a final irrigation of 5 mL NaOCl after mechanical instrumentation. With this approach, we aimed to facilitate the penetration of the solution in diverse directions. The sole distinction among the case groups lies in the employed chelating agents, namely EDTA and HEDP. 

To conduct 3D image analysis, Python was utilized to evaluate micro-CT images captured from entire roots with a 50-voxel thickness encompassing the entire root canal, without the need for sectioning.

In an investigation conducted by Elbahary et al. [11], three slices with thicknesses of 1 mm were excised beneath the CEJ. These slices were submerged in a specified solution and a surface measuring 160 × 160 µm at the midpoint of the bucco-lingual axis was solely chosen for the 3D analysis. Kfir et al. [3] reported that after SEM evaluation on split teeth, there were no substantial distinctions observed in terms of cleanliness, debris presence, smear layer, and canal wall erosion between the group treated with HEDP containing 3% sodium hypochlorite and the group subjected to 3% sodium hypochlorite irrigation followed by 17% EDTA. Kfir et al. assessed the erosion in SEM images and quantified the findings using a three-grade scoring system, encompassing minimal, moderate, and severe levels of erosion [3,53], while we used Python for 3D analysis to assess density and ImageJ2 software to measure 2D erosion depth in micro-CT images. Different methodologies to assess erosion could affect the precision of results and be the reason for differences. 

As per the findings by Li et al. [38], variations in NaOCl concentrations were observed to impact the compressive strength of treated root canals. The study highlighted that the concentrations of NaOCl exceeding 1% influenced the strength of the dentinal wall in the treated roots [38]. Xu et al.’s investigation revealed that the concentration of NaOCl holds greater significance in inducing microstructural and fracture strength changes compared with the concentration of EDTA [13]. This observation could potentially explain the occurrence of erosion in control groups in our study, where irrigation solely involved the application of NaOCl.

**Figure 5:**
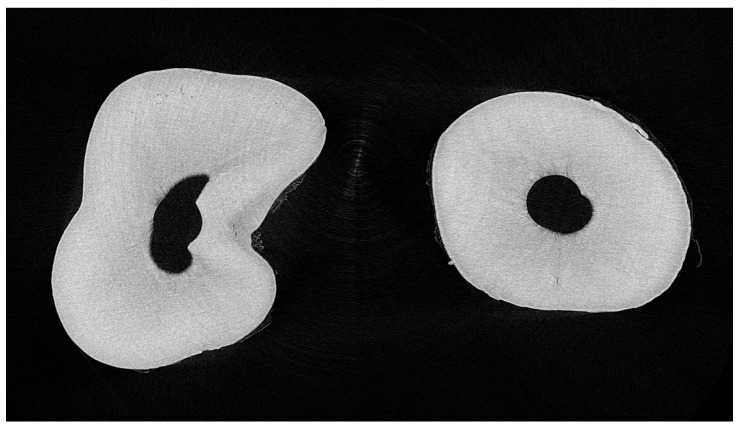
The middle layer of the cervical third in the EDTA group shows changes in root canal morphology made via the ultrasonic instrument.

The duration of irrigations is an additional factor that could influence the strength and fracture resistance of treated roots, as highlighted in previous studies [39,54,55]. In the current investigation, this variable was not taken into account, preventing a direct comparison between our results and those reported in prior studies. Concerning the impact of activating irrigation solutions, Ulusoy et al. investigated the effects of 9% HEBP and 17% EDTA when used with two distinct irrigation systems. Their findings indicated that teeth treated with HEBP and subjected to passive ultrasonic irrigation exhibited greater resistance to fracture in compressive strength tests compared with teeth treated with conventional syringe irrigation and EDTA [46]. However, in another study, it was reported that passive ultrasonic irrigation could increase the destructive effect of NaOCl [56]. In some samples of the EDTA group, the root dentine morphology was changed not only by erosion but also by the ultrasonic instrument touching the inner dentine wall (Figure 5).

Concerning the ultrasonically activated combination of NaOCl and HEDP solution, reports suggest an acceleration of chemical interaction, potentially leading to a quicker loss of the effectiveness of NaOCl [15,57]. This is in agreement with the study of Ballal et al., which demonstrated the synergistic effect between NaOCl and ultrasound. In addition, no interference of HEDP with ultrasonic activation of NaOCl was reported [58].

Several studies examined the efficacy of XP-Endo finisher and passive ultrasonic irrigation in conjunction with either EDTA or HEDP in eliminating the smear layer. Through SEM analysis on single-rooted human teeth, these studies revealed that neither the XP-Endo finisher nor passive ultrasonic irrigation achieved complete removal of the smear layer. Notably, passively ultrasonically activated 17% EDTA emerged as the most effective irrigation method for smear layer removal [49]. Our study differs from others in that we employed non-destructive visualization and measurement of erosion using the IrriS VDW Ultra motor at a 20% setting in both the EDTA and HEDP groups. It is important to note that we cannot further discuss the effectiveness of ultrasonic irrigation in our study, as we did not evaluate the impact of activated irrigation in various groups.

Not considering the total duration of canal preparation and irrigation is one of the limitations of this study. As the erosive effect is time- and concentration-dependent, further studies should consider factors such as the application and activation time, the total volume of irrigants, and the irrigation protocol.

The possibility of EDTA penetration into the control roots during ultrasonic activation could be a weakness of this study. Consequently, it is recommended to use composite adhesive for sealing in future studies. 

In this study, as with many light microscopy studies, a single slice was selected from the central region of each root third for 2D measurements. This could be a limitation, as, theoretically, it would have been possible to measure in approximately 978 slices per third. However, due to time and cost constraints, this extensive approach was not undertaken. 

## 5. Conclusions

HEDP offers a solution to the compatibility issue between EDTA and NaOCl. Implementing minimally invasive irrigation methods alongside chelating agents can minimize erosion, which may lessen the potential of vertical root fracture and adverse effects on the mechanical strength and micro-hardness of dentin, which could reduce the longevity of the treated tooth [3].

## Figures and Tables

**Table 1:** Descriptive summary of mean erosion depth grouped by position, group, and chelating agent.

Group	Position	Mean	SD	Min	Q1	Median	Q3	Max
EDTA—Case	Cervical	45.75	10.25	31.71	35.45	49.72	51.55	62.32
	Middle	41.79	10.10	26.58	34.41	41.08	48.96	58.79
	Apical	32.25	12.52	16.09	22.41	30.04	38.36	52.12
EDTA—Control	Cervical	15.78	8.20	4.79	11.21	14.27	22.14	29.89
	Middle	14.43	8.30	3.07	8.47	15.26	17.29	31.31
	Apical	12.19	7.16	1.58	8.14	12.72	16.85	22.53
HEDP—Case	Cervical	20.25	8.53	8.21	15.53	18.79	21.83	38.46
	Middle	16.40	11.05	4.23	8.68	11.33	21.99	38.45
	Apical	15.96	11.97	3.46	6.18	11.17	25.23	38.69
HEDP—Control	Cervical	11.82	5.65	5.68	8.23	10.25	12.58	23.66
	Middle	12.98	10.45	3.98	6.00	7.64	16.49	35.94
	Apical	10.61	6.84	3.86	5.63	8.03	13.75	25.39

**Table 2:** Summary of the repeated-measures ANOVA (Example 1).

Repeated Measures ANOVA	Repeated Measurement Factors	*p* Value
One-way interaction	Chelating agent (EDTA/HEDP)	0.004
	Group (Case/Control)	<0.0001
	Position (Cervical, Middle, Apical)	0.002
Two-way interactions	Chelating agent (EDTA/HEDP): Group (Case/Control)	<0.0001
	Position (Cervical, Middle, Apical): Group (Case/Control)	0.17
	Chelating agent (EDTA/HEDP): Position (Cervical, Middle, Apical)	0.64
Three-way interactions	Chelating agent (EDTA/HEDP): Group (Case/Control): Position (Cervical, Middle, Apical)	0.20

**Table 3:** Summary of the repeated-measures ANOVA (Example 2).

Repeated Measures ANOVA	Repeated Measurement Factors	*p* Value
One-way interaction	Chelating agent (EDTA/HEDP)	<0.0001
	Group (Case/Control)	0.01
	Position (Cervical, Middle, Apical)	0.31
Two-way interactions	Chelating agent (EDTA/HEDP): Group (Case/Control)	0.12
	Position (Cervical, Middle, Apical): Group (Case/Control)	0.89
	Chelating agent (EDTA/HEDP): Position (Cervical, Middle, Apical)	0.055
Three-way interactions	Chelating agent (EDTA/HEDP): Group (Case/Control): Position (Cervical, Middle, Apical)	0.17

## Data Availability

Not applicable.

## References

[1] Eick J.D., Wilko R.A., Anderson C.H., Sorensen S.E. (1970). Scanning electron microscopy of cut tooth surfaces and identification of debris by use of the electron microprobe. J. Dent. Res..

[2] McComb D., Smith D.C. (1975). A preliminary scanning electron microscopic study of root canals after endodontic procedures. J. Endod..

[3] Kfir A., Goldenberg C., Metzger Z., Hülsmann M., Baxter S. (2020). Cleanliness and erosion of root canal walls after irrigation with a new HEDP-based solution vs. traditional sodium hypochlorite followed by EDTA. A scanning electron microscope study. Clin. Oral Investig..

[4] Ulusoy Ö.İ., Mantı A.Ş., Çelik B. (2020). Nanohardness reduction and root dentine erosion after final irrigation with ethylenediaminetetraacetic, etidronic and peracetic acids. Int. Endod. J..

[5] Zehnder M. (2006). Root canal irrigants. J. Endod..

[6] Spratt D.A., Pratten J., Wilson M., Gulabivala K. (2001). An in vitro evaluation of the antimicrobial efficacy of irrigants on biofilms of root canal isolates. Int. Endod. J..

[7] Naenni N., Thoma K., Zehnder M. (2004). Soft tissue dissolution capacity of currently used and potential endodontic irrigants. J. Endod..

[8] Yadav H.K., Yadav R.K., Chandra A., Tikku A.P. (2017). A Scanning Electron Microscopic Evaluation of the Effectiveness of Etidronic Acid, SmearClear and MTAD in Removing the Intracanal Smear Layer. J. Dent..

[9] Lottanti S., Gautschi H., Sener B., Zehnder M. (2009). Effects of ethylenediaminetetraacetic, etidronic and peracetic acid irrigation on human root dentine and the smear layer. Int. Endod. J..

[10] Violich D.R., Chandler N.P. (2010). The smear layer in endodontics—A review. Int. Endod. J..

[11] Elbahary S., Haj-Yahya S., Khawalid M., Tsesis I., Rosen E., Habashi W., Pokhojaev A., Sarig R. (2020). Effects of different irrigation protocols on dentin surfaces as revealed through quantitative 3D surface texture analysis. Sci. Rep..

[12] Rosatto C.M.P., Ferraz D.C., Oliveira L.V., Soares P.B.F., Soares C.J., Tanomaru Filho M., Moura C.C.G. (2021). Effect of irrigation protocols on root canal wall after post preparation: A micro-CT and microhardness study. Braz. Oral Res..

[13] Xu H., Ye Z., Zhang A., Lin F., Fu J., Fok A.S.L. (2022). Effects of concentration of sodium hypochlorite as an endodontic irrigant on the mechanical and structural properties of root dentine: A laboratory study. Int. Endod. J..

[14] Wright P.P., Scott S., Kahler B., Walsh L.J. (2020). Organic tissue dissolution in clodronate and etidronate mixtures with sodium hypochlorite. J. Endod..

[15] Zollinger A., Mohn D., Zeltner M., Zehnder M. (2018). Short-term storage stability of NaOCl solutions when combined with Dual Rinse HEDP. Int. Endod. J..

[16] Yamada R.S., Armas A., Goldman M., Lin P.S. (1983). A scanning electron microscopic comparison of a high volume final flush with several irrigating solutions: Part 3. J. Endod..

[17] Gómez-Delgado M., Camps-Font O., Luz L., Sanz D., Mercade M. (2023). Update on citric acid use in endodontic treatment: A systematic review. Odontology.

[18] Berry E.A., von der Lehr W.N., Herrin H.K. (1987). Dentin surface treatments for the removal of the smear layer: An SEM study. J. Am. Dent. Assoc..

[19] McComb D., Smith D.C., Beagrie G.S. (1976). The results of in vivo endodontic chemomechanical instrumentation—A scanning electron microscopic study. J. Br. Endod. Soc..

[20] Bitter N.C. (1989). A 25% tannic acid solution as a root canal irrigant cleanser: A scanning electron microscope study. Oral Surg. Oral Med. Oral Pathol..

[21] Sabbak S.A., Hassanin M.B. (1998). A scanning electron microscopic study of tooth surface changes induced by tannic acid. J. Prosthet. Dent..

[22] Meryon S.D., Tobias R.S., Jakeman K.J. (1987). Smear removal agents: A quantitative study in vivo and in vitro. J. Prosthet. Dent..

[23] Grawehr M., Sener B., Waltimo T., Zehnder M. (2003). Interactions of ethylenediamine tetraacetic acid with sodium hypochlorite in aqueous solutions. Int. Endod. J..

[24] Zehnder M., Schmidlin P., Sener B., Waltimo T. (2005). Chelation in root canal therapy reconsidered. J. Endod..

[25] Patil P.H., Gulve M.N., Kolhe S.J., Samuel R.M., Aher G.B. (2018). Efficacy of new irrigating solution on smear layer removal in apical third of root canal: A scanning electron microscope study. J. Conserv. Dent..

[26] Rossi-Fedele G., Doğramaci E.J., Guastalli A.R., Steier L., de Figueiredo J.A. (2012). Antagonistic interactions between sodium hypochlorite, chlorhexidine, EDTA, and citric acid. J. Endod..

[27] Nogo-Živanović D., Kanjevac T., Bjelović L., Ristić V., Tanasković I. (2019). The effect of final irrigation with MTAD, QMix, and EDTA on smear layer removal and mineral content of root canal dentin. Microsc. Res. Tech..

[28] Ulusoy Ö.İ., Görgül G. (2013). Effects of different irrigation solutions on root dentine microhardness, smear layer removal and erosion. Aust. Endod. J..

[29] Biel P., Mohn D., Attin T., Zehnder M. (2017). Interactions between the Tetrasodium Salts of EDTA and 1-Hydroxyethane 1,1-Diphosphonic Acid with Sodium Hypochlorite Irrigants. J. Endod..

[30] Neelakantan P., Varughese A.A., Sharma S., Subbarao C.V., Zehnder M., De-Deus G. (2012). Continuous chelation irrigation improves the adhesion of epoxy resin-based root canal sealer to root dentine. Int. Endod. J..

[31] Paqué F., Rechenberg D.K., Zehnder M. (2012). Reduction of hard-tissue debris accumulation during rotary root canal instrumentation by etidronic acid in a sodium hypochlorite irrigant. J. Endod..

[32] Morago A., Ordinola-Zapata R., Ferrer-Luque C.M., Baca P., Ruiz-Linares M., Arias-Moliz M.T. (2016). Influence of Smear Layer on the Antimicrobial Activity of a Sodium Hypochlorite/Etidronic Acid Irrigating Solution in Infected Dentin. J. Endod..

[33] Neelakantan P., Cheng C.Q., Mohanraj R., Sriraman P., Subbarao C., Sharma S. (2015). Antibiofilm activity of three irrigation protocols activated by ultrasonic, diode laser or Er:YAG laser in vitro. Int. Endod. J..

[34] Tartari T., Bachmann L., Zancan R.F., Vivan R.R., Duarte M.A.H., Bramante C.M. (2018). Analysis of the effects of several decalcifying agents alone and in combination with sodium hypochlorite on the chemical composition of dentine. Int. Endod. J..

[35] Aranda-Garcia A.J., Kuga M.C., Chavéz-Andrade G.M., Kalatzis-Sousa N.G., Hungaro Duarte M.A., Faria G., Reis Só M.V., Faria N.B. (2013). Effect of final irrigation protocols on microhardness and erosion of root canal dentin. Microsc. Res. Tech..

[36] Pascon F.M., Kantovitz K.R., Soares L.E., Santo A.M., Martin A.A., Puppin-Rontani R.M. (2012). Morphological and chemical changes in dentin after using endodontic agents: Fourier transform Raman spectroscopy, energy-dispersive x-ray fluorescence spectrometry, and scanning electron microscopy study. J. Biomed. Opt..

[37] Brännström M., Johnson G. (1974). Effects of various conditioners and cleaning agents on prepared dentin surfaces: A scanning electron microscopic investigation. J. Prosthet. Dent..

[38] Li A.L.B., Markvart M., Abbott P.V. (2022). Effect of Different Concentrations of Sodium Hypochlorite on the Compressive Strength of Endodontically Treated Roots. J. Endod..

[39] Gonzalez C.S., Estevez R., Loroño G., García V.D., Caballero Montes J.A., Rossi-Fedele G., Cisneros R. (2020). Etidronic acid and ethylenediaminetetraacetic acid associated with sodium hypochlorite have limited effect on the compressive fracture resistance of roots ex vivo. J. Conserv. Dent..

[40] Zarean P., Özcan M., Zarean P., Haghani S.O., Jahromi M.Z., Al-Haj Husain N., Khabiri M. (2022). Micro-Computed Tomographic Assessment of Microcrack Formation before and after Instrumentation of Curved Root Canals with Neoniti Rotary Files. Materials.

[41] Cassimiro M., Romeiro K., Gominho L., de Almeida A., Costa L., Albuquerque D. (2017). Occurence of dentinal defects after root canal preparation with R-phase, M-Wire and Gold Wire instruments: A micro-CT analysis. BMC Oral Health.

[42] Cai M., Cai Y., Yang R., Xu Z., Neelakantan P., Wei X. (2022). Impact of agitation/activation strategies on the antibiofilm potential of sodium hypochlorite/etidronate mixture in vitro. BMC Oral Health.

[43] R Core Team (2020). A Language and Environment for Statistical Computing.

[44] Brunner E., Puri M. (2001). Nonparametric Methods in Factorial Designs.

[45] Basandi P.S., Madammal R.M., Adi R.P., Donoghue M., Nayak S., Manickam S. (2015). Predentin thickness analysis in developing and developed permanent teeth. J. Nat. Sci. Biol. Med..

[46] Ulusoy Ö.İ., Genç Şen Ö., Zeyrek S., Kaya M., Paltun Y.N. (2021). Effect of final irrigation protocols on the fracture resistance of roots with varying dentine thickness. Eur. J. Oral Sci..

[47] Russell A.A., Chris He L.H., Chandler N.P. (2014). Investigation of dentin hardness in roots exhibiting the butterfly effect. J. Endod..

[48] Zehnder M., Schicht O., Sener B., Schmidlin P. (2005). Reducing surface tension in endodontic chelator solutions has no effect on their ability to remove calcium from instrumented root canals. J. Endod..

[49] Espinoza I., Conde Villar A.J., Loroño G., Estevez R., Plotino G., Cisneros R. (2021). Effectiveness of XP-Endo Finisher and Passive Ultrasonic Irrigation in the Removal of the Smear Layer Using two Different Chelating Agents. J. Dent..

[50] Kamel W.H., Kataia E.M. (2014). Comparison of the efficacy of Smear Clear with and without a canal brush in smear layer and debris removal from instrumented root canal using WaveOne versus ProTaper: A scanning electron microscopic study. J. Endod..

[51] Fraser J.G. (1974). Chelating agents: Their softening effect on root canal dentin. Oral Surg. Oral Med. Oral Pathol..

[52] Wang Z., Maezono H., Shen Y., Haapasalo M. (2016). Evaluation of root canal dentin erosion after different irrigation methods using energy-dispersive X-ray spectroscopy. J. Endod..

[53] Kaya S., Yiğit-Özer S., Adigüzel Ö. (2011). Evaluation of radicular dentin erosion and smear layer removal capacity of Self-Adjusting File using different concentrations of sodium hypochlorite as an initial irrigant. Oral Surg. Oral Med. Oral Pathol. Oral Radiol. Endod..

[54] Uzunoglu E., Aktemur S., Uyanik M.O., Durmaz V., Nagas E. (2012). Effect of ethylenediaminetetraacetic acid on root fracture with respect to concentration at different time exposures. J. Endod..

[55] Bhandary S., Kakamari S., Srinivasan R., Chandrappa M.M., Nasreen F., Junjanna P. (2017). A comparative evaluation of the effect of 8% and 17% ethylenediaminetetraacetic acid exposure for 1 min and 10 min on the fracture resistance of endodontically treated roots: An in vitro study. J. Conserv. Dent..

[56] Osiri S., Banomyong D., Sattabanasuk V., Yanpiset K. (2018). Root rein- forcement after obturation with calcium silicate-based sealer and modified gutta-percha cone. J. Endod..

[57] Wright P.P., Kahler B., Walsh L.J. (2019). The Effect of Heating to Intracanal Temperature on the Stability of Sodium Hypochlorite Admixed with Etidronate or EDTA for Continuous Chelation. J. Endod..

[58] Ballal N.V., Ivica A., Meneses P., Narkedamalli R.K., Attin T., Zehnder M. (2021). Influence of 1-Hydroxyethylidene-1,1-Diphosphonic Acid on the Soft Tissue-Dissolving and Gelatinolytic Effect of Ultrasonically Activated Sodium Hypochlorite in Simulated Endodontic Environments. Materials.

